# CTNNB1 Alternation Is a Potential Biomarker for Immunotherapy Prognosis in Patients With Hepatocellular Carcinoma

**DOI:** 10.3389/fimmu.2021.759565

**Published:** 2021-10-28

**Authors:** Lin Chen, Qiaodan Zhou, Junjie Liu, Wei Zhang

**Affiliations:** ^1^ Hepatic Surgery Center, Tongji Hospital, Tongji Medical College, Huazhong University of Science and Technology, Wuhan, China; ^2^ Department of Nephrology, Tongji Hospital, Tongji Medical College, Huazhong University of Science and Technology, Wuhan, China

**Keywords:** hepatocellular carcinoma, immune checkpoint inhibitors, prognosis, biomarker, CTNNB1

## Abstract

**Background:**

The emergence of immune checkpoint inhibitors (ICIs) marks the beginning of a new era of immunotherapy for hepatocellular carcinoma (HCC), however, not all patients respond successfully to this treatment. A major challenge for HCC immunotherapy is the development of ways to screen for those patients that would benefit from this type of treatment and determine the optimal treatment plan for individual patients. Therefore, it is important to find a biomarker which allows for the stratification of HCC patients, which distinguishes responders from non-responders, thereby further improving the clinical benefits for those undergoing immunotherapy.

**Methods:**

We used univariate and multivariate Cox risk proportional regression models to evaluate the relationship between non-synonymous mutations with a mutation frequency greater than 10%. We made a prognosis of an immunotherapy HCC cohort using mutation and prognosis data. An additional three HCC queues from the cbioportal webtool were used for further verification. The CIBERSORT, IPS, quanTIseq, and MCPcounter algorithms were used to evaluate the immune cells. PCA and z-score algorithm were used to calculate immune-related signature with published gene sets. Gene set enrichment analysis (GSEA) was used to compare the differences in the pathway-based enrichment scores of candidate genes between mutant and wild types.

**Results:**

Univariate and multivariate Cox results showed that only CTNNB1-Mutant(CTNNB1-MUT) was associated with progression-free survival (PFS) of HCC patients in the immunotherapy cohort. After excluding the potential bias introduced by other clinical features, it was found that CTNNB1-MUT served as an independent predictor of the prognosis of HCC patients after immunotherapy (P < 0.05; HR > 1). The results of the tumor immune microenvironment (TIME) analysis showed that patients with CTNNB1-MUT had significantly reduced activated immune cells [such as T cells, B cells, M1-type macrophages, and dendritic cells (DCs)], significantly increased M2-type macrophages, a significantly decreased expression of immunostimulating molecules, low activity of the immune activation pathways (cytokine pathway, immune cell activation and recruitment) and highly active immune depletion pathways (fatty acid metabolism, cholesterol metabolism, and Wnt pathway).

**Conclusions:**

In this study, we found CTNNB1-MUT to be a potential biomarker for HCC immunotherapy patients, because it identified those patients are less likely to benefit from ICIs.

## Introduction

Hepatocellular carcinoma (HCC) accounts for 75-85% of primary liver cancers, ranking sixth among the most common cancers, globally. It ranks fourth among cancer-related deaths, and has a morbidity and mortality of 4.7% and 8.2% (1), respectively. For about 60% of HCC patients surgery is no longer an option at the time of diagnosis, and the 5-year survival rate is 12.5% (2–4). Previously, the main treatment method for advanced HCC was targeted therapy and systemic chemotherapy, however, this has shown limited efficacy (5). At present, treatments involving the use of immune checkpoint inhibitors (ICIs), such as those which target the programmed death receptor-1/programmed death ligand-1 (PD-1/PD-L1) pathway, are currently a priority for research in the field of advanced HCC (6, 7). CheckMate-040 and Keynote-224 resulted in accelerated FDA approval of Nivolumab and Pembrolizumab as single drugs for second-line treatment of advanced HCC, and opened a new era of immunotherapy for HCC (6, 7). Nevertheless, there are still many patients who do not respond to immunotherapy alone. Thus, the ability to screen for the patients that would benefit and determine the best combination therapy for individual patients has become a major challenge for the treatment of HCC.

An increasing number of studies have begun looking for biomarkers related to the curative effect, with the aim of stratifying HCC patients and distinguishing responders from non-responders. To avoid unnecessary toxicity, alternative treatments are recommended for patients who are not expected to respond to immunotherapy (8, 9). Existing biomarkers include PD-L1 expression, tumor mutation burden (TMB), tumor-infiltrating lymphocytes (TILs), and cytokines (8–14). The classification of PD-L1 in HCC is complex, and the level of spatial and cellular heterogeneity is high, which may affect the reliability and repeatability of PD-L1 as a predictor of response to treatment with ICIs in comparison to other markers (15). The heterogeneity of these markers in different laboratories and platforms, and dynamic changes of immune cells and cytokines remains a challenge to using them to predict the prognosis of HCC patients after immunotherapy (12). Therefore, it is extremely important to screen and evaluate the efficacy of a larger variety of immune therapy-related biomarkers including ICIs.

Specific gene mutations can also be used as a biomarker to predict the response to treatment with ICIs (10, 16–20). Harding et al. performed prospective sequencing on HCC patients who received ICIs and found that of the 10 patients with the Wnt-pathway mutation, the disease had progressed at the first interval scan, representing no clinical response to treatment. However, nine (53%) of 17 patients without the Wnt-pathway mutation had long-lasting stability (≥4 months) or improved clinical efficacy, indicating that the Wnt-pathway mutation may be an effective biomarker for predicting the response of patients with HCC to treatment with ICI (17). Other studies have shown that the TERT promoter, co-mutation of bromine-containing domain protein 4 (BRD4), and tumor protein P53 (TP53) can also be used as reliable signals to predict mutation risk, thus guiding the selection of the treatment regimen for individualized HCC immunotherapy (16). With the current progress of clinical trials for HCC immunotherapy, the relationship between systematic genetic screening and the clinical prognosis of HCC patients after receiving ICIs has not yet been confirmed. Therefore, we collected a cohort of HCC patients with ICIs as part of their treatment plan from an open database, conducted further comprehensive screening for ICI-related biomarkers, and then studied the relationship between the TIME and the candidate biomarkers. Through this work, we hope to improve the clinical application of immunotherapy in patients with HCC by allowing for a more accurate selection of which patients are most likely to respond to treatment.

## Methods

### HCC Cohort

To explore the relationship between gene mutation and clinical prognosis of HCC patients after immunotherapy, we collected a cohort of HCC patients receiving ICIs (Harding et.al.) *via* the cbioportal (21) web tool (designated Harding-HCC) (17). For this cohort we downloaded targeted sequencing data and the clinical data of all HCC patients. To further explore the predictive role of gene mutations in other HCC cohorts, we collected three more from the cbioportal web tool [Xue-HCC (22), Ahn-HCC (23) and TCGA-LIHC (24)] which included whole exome sequencing (WES) and clinical data.

### Screening for Mutation-Based Predictive Markers

For the Harding-HCC cohort, we first filtered the synonymous mutations according to the definition and classification of non-synonymous mutations using the R package ‘maftools’, and finally kept only the non-synonymous mutation data (25). We then screened for non-synonymous mutations with a mutation frequency greater than 10%, as genes with low mutation rates are a source of potential bias in subsequent analyses. These mutations were used for downstream analysis. Univariate and multivariate COX regression models were used to screen genes with significant predictive effect on the prognosis of HCC patients undergoing immunotherapy, with P < 0.05 regarded as statistically significant. Additionally, we took the following steps to eliminate any bias introduced by common clinical factors when predicting the prognosis of immunotherapy. Among the screened candidate mutant genes, we added common clinical factors to the univariate and multivariate COX regression models and controlled for any bias introduced by these clinical characteristics when predicting the prognosis of immunotherapy.

### TIME Analysis

The algorithms used for immune cell analysis were CIBERSORT, IPS, quanTIseq, and MCP counter. Based on these algorithms (26–29), we were able to obtain the relevant scores of the infiltration of immune cells in the TIME based on the expression data. Immunostimulation, immunosuppression, immune checkpoint molecules and major histocompatibility complex (MHC)-related genes were derived from the results published by Charoentong and his colleagues (29). We collected the immune-related gene sets from published studies and used the PCA and z-score algorithms to score each patient in relation to their immune-related signature. Gene set enrichment analysis (GSEA) was used to compare the difference in expression between mutant and wild type candidate genes, and to calculate the pathway scores and p values from the GO, KEGG and Reactome databases (30).

### Drug Sensitivity Analysis

On the basis of 138 drugs in the genomics of drug sensitivity in cancer (GDSC) database (31), the half maximum inhibition concentration (IC50) for each patient in the TCGA-LIHC cohort was estimated by ridge regression method. This was based on the expression data of the TCGA-LIHC cohort determined *via* the R package ‘pRRophetic (32)’.

### Statistical Analysis

**Figure 1:**
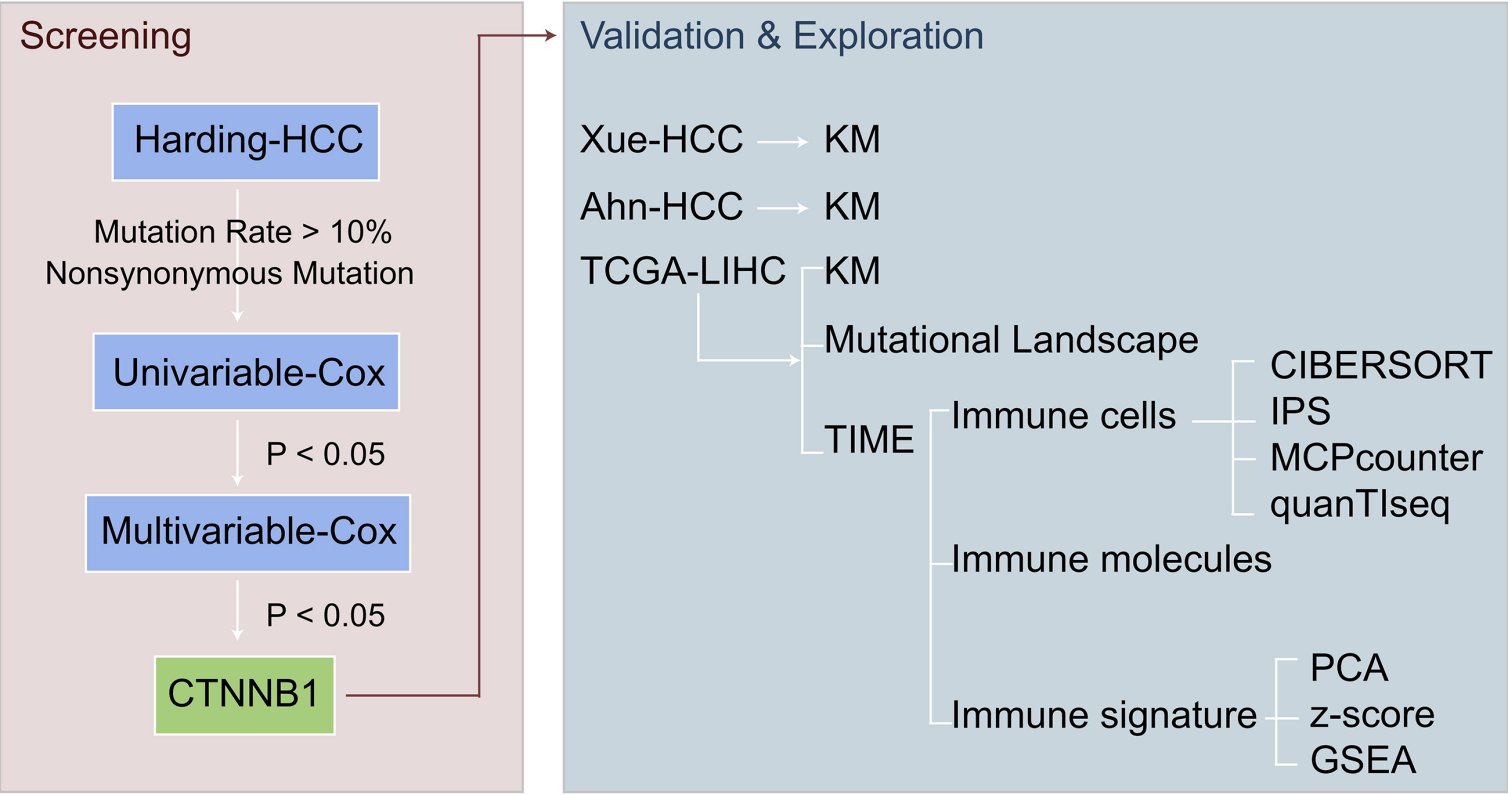
Overview of the screening for potential biomarkers of HCC patients with ICI treatment.

For this study, a univariate Cox proportional risk regression model was used to calculate the risk ratio of the univariate analysis, and a multivariate Cox regression model was used to determine the independent prognostic factors. For the survival analysis of HCC patients, the log rank Pvalue, hazard ratio, and 95% confidence intervals (CI) were calculated using the R packages ‘survival’ and ‘survminer.’ First, a Shapiro-Wilk normality test was carried out to test the statistical significance of the normal distribution of the variables (33), and as the immune-related scores in this study were not normally distributed, we used a Mann-Whitney U test to analyze the differences in the immune-related scores between the mutant and wild types of the candidate genes. We used Fisher’s exact test to compare the mutation frequency between the mutant and wild type candidate genes. In addition, we also used the imperial Bayes test statistics methods from the R package ‘Limma’ to compare the expression of immune cells or immune-related molecules between the mutant and wild type candidate genes, and to calculate the log fold change (logFC) and P value. The ‘somaticInteractions’ function of the maftools was used to performs pair-wise fisher’s exact test to detect mutually exclusive or co-occurring events. In this study, all data analyses and visualizations were completed using the software R (Version. 3.6). The P value is bilateral with less than 0.05 considered to be statistically different. See **Figure 1** for an overview of the flow of the research presented in this paper.

## Results

### CTNNB1 Can Be Used as an Independent Predictor of the Prognosis of HCC Patients After Immunotherapy

**Figure 2:**
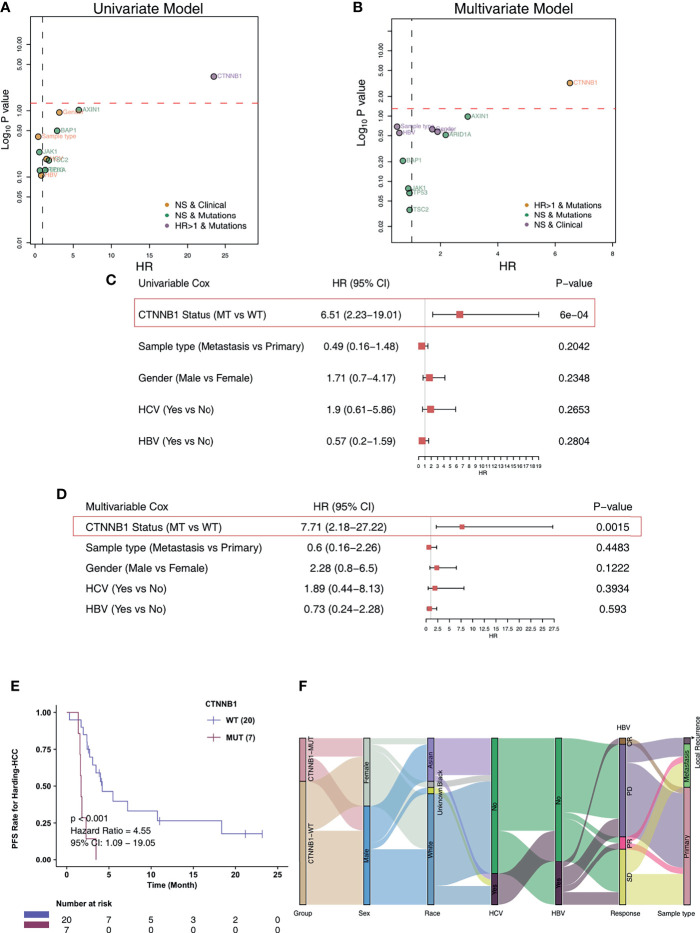
CTNNB1-MUT was associated with shorter PFS in HCC patients responding to ICI treatment. Univariable Cox regression analysis **(A)** and multivariable Cox regression analysis **(B)** in subgroups of mutation, sample type, HBV status, HCV status, and gender in the Harding-HCC cohort. Univariable Cox regression analysis **(C)** and multivariable Cox regression analysis **(D)** in subgroups of CTNNB1 mutation status, sample type, HBV status, HCV status, and gender in the Harding-HCC cohort. **(E)** Kaplan-Meier curves comparing the progression-free survival (PFS) of patients with CTNNB1-Mutant (CTNNB1-MUT) and patients with CTNNB1-wildtype (CTNNB1-WT) in the Harding-HCC cohort. **(F)** A Sankey diagram visualizing the clinical characteristics of CTNNB1-MUT and CTNNB1-WT patients in the Harding-HCC cohort.

For the Harding-HCC cohort, we constructed a univariate Cox proportional hazard regression model for the prognosis of patients with non-synonymous mutations and with mutation frequencies greater than 10%. The results showed that only CTNNB1-Mutant (CTNNB1-MUT) was related to a less favorable prognosis for HCC patients (HR = 6.51, P = 0.0006; **Figure 2A**). Following this, the multivariate Cox proportional hazard regression model we constructed showed that CTNNB1-MUT could be used as an independent predictor of immunotherapy response in patients with HCC (HR = 7.71, P = 0.002; **Figure 2B**). To further eliminate the potential bias caused by some common clinical variables in the risk ratio regression model, we performed a combined analysis of CTNNB1-MUT and covariates (such as sample type, gender, HCV, and HBV). The results showed that, in both the univariate and multivariate Cox risk ratio model, only CTNNB1-MUT is significantly associated with a worse clinical prognosis for HCC patients (all P < 0.05; HR >1; **Figures 2C, D**). The analysis of survival among this cohort showed that the PFS benefit was more significant for those in the CTNNB1-WT group compared to those in the CTNNB1-MUT group (log rank p < 0.001, HR = 4.55, 95%CI: 1.09-19.05; **Figure 2E**). **Figure 2F** and **Table S1** show the distribution of CTNNB1-MUT and CTNNB1-wildtype (CTNNB1-WT) along with the clinical features of the patients in the Harding-HCC cohort.

**Figure 3:**
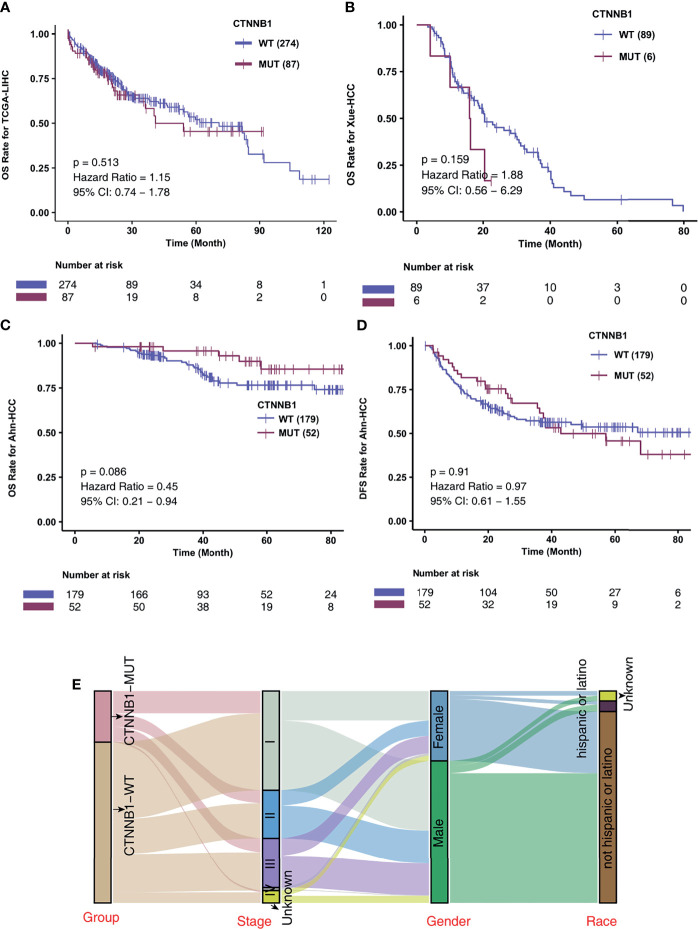
CTNNB1-MUT was not associated with prognosis in HCC patients responding to routine treatment. Kaplan-Meier curves comparing the overall survival (OS) of patients with CTNNB1-Mutant (CTNNB1-MUT) and patients with CTNNB1-wildtype (CTNNB1-WT) in the TCGA-LIHC **(A)**, Xue-HCC **(B)** and Ahn-HCC **(C)** cohorts. **(D)** Kaplan-Meier curves comparing the disease-free survival (DFS) of patients with CTNNB1-Mutant (CTNNB1-MUT) and patients with CTNNB1-wildtype (CTNNB1-WT) in the Ahn-HCC cohort. **(E)** A Sankey diagram visualizing the clinical characteristics between CTNNB1-MUT and CTNNB1-WT patients in the TCGA-LIHC cohort.

To investigate the association of CTNNB1-MUT on the prognosis of HCC patients after conventional treatment, we further calculated the difference in survival of patients between CTNNB1-MUT and CTNNB1-WT in three non-ICI-treated HCC cohorts. A difference in survival was not observed for these cohorts: TCGA-LIHC cohort (log rank P = 0.514; **Figure 3A**), Xue-HCC cohort (log rank P = 0.159; **Figure 3B**), Ahn-HCC-OS cohort (log rank P = 0.086; **Figure 3C**), and Ahn-HCC-DFS cohort (log rank P = 0.91; **Figure 3D**). **Figure 3E** and **Table S2** show the distribution of CTNNB1-MUT and CTNNB1-WT along with the clinical features of the patients in the TCGA-LIHC cohort.

### Overall Difference in Gene Mutations Between CTNNB1-MUT and CTNNB1-WT

**Figure 4:**
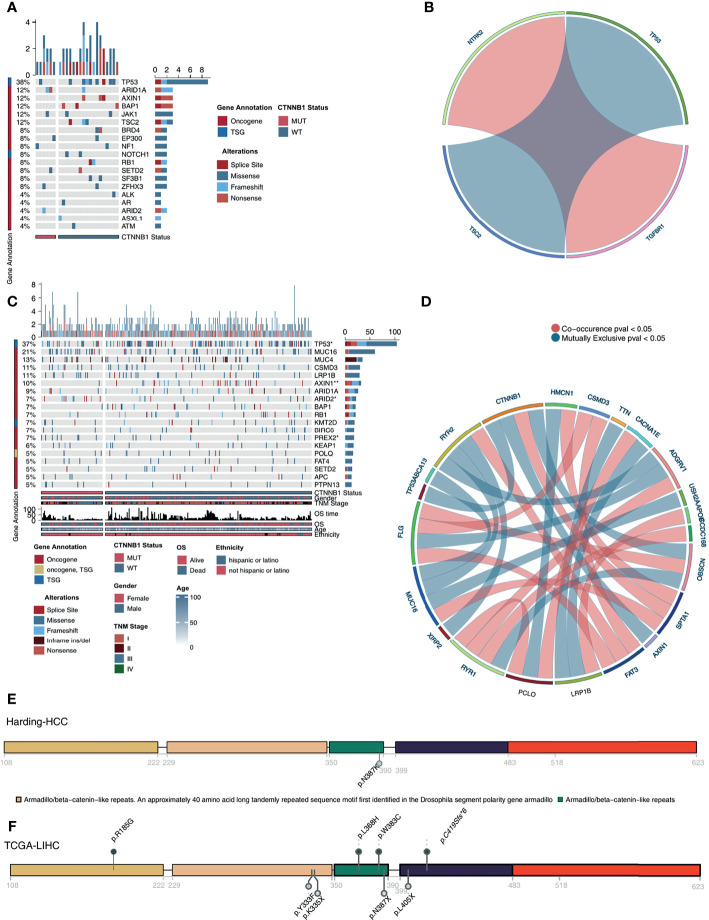
Genomic profiles of HCC patients. **(A)**. The top 20 mutated driver genes in the Harding-HCC cohort. **(B)**. Ribbon plot showing the co-occurrence (or mutually exclusive relation) between pairs of mutated driver genes in the Harding-HCC cohort. **(C)** The top 20 mutated driver genes in the TCGA-LIHC cohort. **(D)** Ribbon plot showing the co-occurrence (or mutually exclusive relation) between pairs of mutated driver genes in the TCGA-LIHC cohort. Lollipop plot showing the distribution of CTNNB1 mutations in the Harding-HCC **(E)** and TCGA-LIHC **(F)** cohorts. *P < 0.05; **P < 0.01.

In the Harding-HCC cohort, we compared and visualized the mutation frequency and types of the driver genes which had a mutation frequency ranking in the top 20 in the CTNNB1-MUT and CNTTB1-WT groups. **Figure 4A** shows that most of these 20 driver genes are oncogenes, with only a few being tumor suppressor genes (TSGs). Missense mutations were the most common mutation type seen in this selection of genes, with the other mutation types (such as splice site, frameshift, and senseless mutation) only accounting for a small proportion. A difference in mutation frequency of the 20 driver genes was not observed in this cohort, and this may be due to the small sample size. The results of mutual exclusion and co-occurrence analysis of the top 20 driver genes is shown in **Figure 4B**. In addition, we also analyzed the difference in mutation frequency of the top 20 genes between the CTNNB1-MUT and CTNNB1-WT groups, and there was no significant difference (all P > 0.05; **Figure S1A**). In the TCGA-LIHC cohort, we found that TP53 (TSG) and AXIN1 (oncogene) had significantly lower mutation frequency in the CTNNB1-MUT group compared to the CTNNB1-WT group (19.54%% *vs*. 31.16% and 1.15% *vs*. 9.78% respectively; p < 0.05; **Figure 4C**) On the other hand, the mutation frequencies of ARID2 (oncogene, 11.49% *vs*. 3.62%) and PREX2 (oncogene, 9.60% *vs*. 3.62%) were significantly higher in the CTNNB1-MUT group compared to the CTNNB1-WT group (P < 0.05; **Figure 4C**). The results of the mutual exclusion and co-occurrence analysis of the top 20 driver genes for the TCGA-LIHC cohort are shown in **Figure 4D**, and the difference in mutation frequency of the top 20 genes in both CTNNB1-MUT and CTNNB1-WT groups are shown in **Figure S1B**. **Figures 4E, F** further shows the mutation sites of CTNNB1 in the Harding-HCC and TCGA-LIHC cohorts. From this, we can see that the majority of mutation sites are located within the armadillo/beta-catenin-like repetitions.

### CTNNB1-MUT Is Related to an Immune-Exhausted TIME

**Figure 5:**
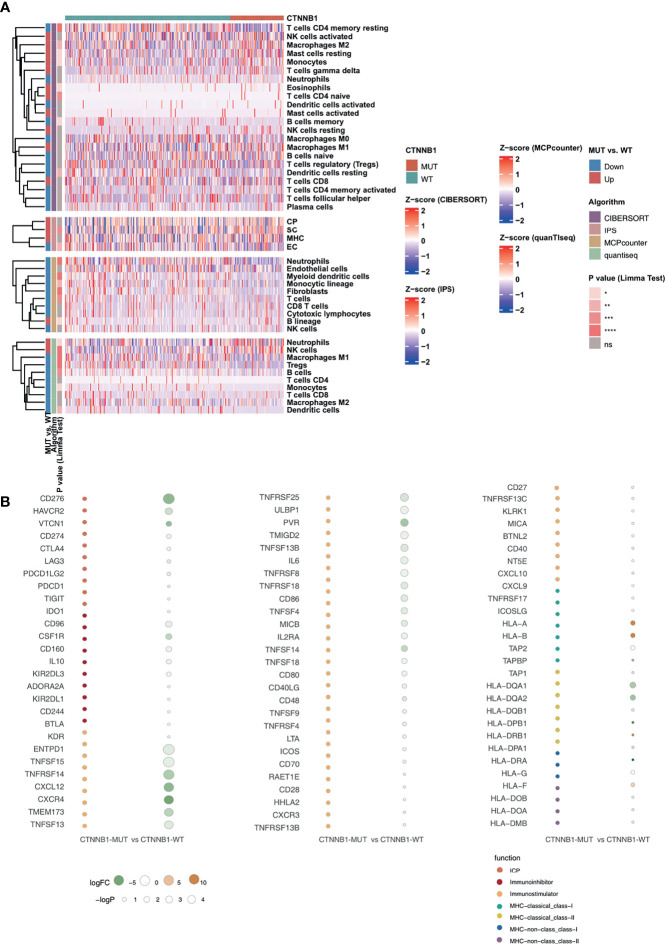
CTNNB1-WT was associated with activated antitumor immunity. **(A)** Landscape of the immune cells for the TCGA-LIHC cohort. Immune cell scores estimated by the CIBERSORT, IPS, MCPcounter, and quanTIseq were analyzed using the Limma. Tobacco smoking history and clinical stage were analyzed using Fisher’s exact test. The corresponding levels of significance, algorithms and trend are displayed as a heatmap in the left panel. **(B)** Bubble plot depicting the mean differences in immune-related gene mRNA expression between CTNNB1-MUT and CTNNB1-WT tumors in the TCGA-LIHC cohort. The x-axis indicates different histological subtypes and the y-axis indicates gene names. The size of the circle represents the difference [-log10(p-value)] of each indicated immune signature or immune-related gene between CTNNB1-MUT and CTNNB1-WT tumors. Orange indicates upregulation, while green indicates downregulation. *P < 0.05; **P < 0.01; ***P < 0.001; ****P < 0.0001; ns, not significant.

**Figure 6:**
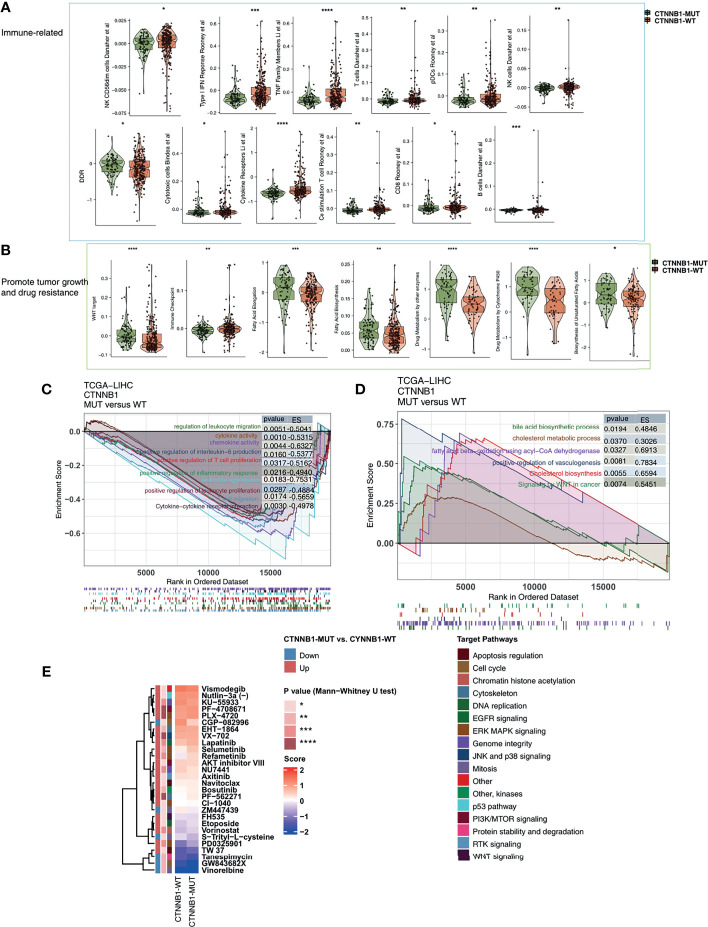
The transcript traits between CTNNB1-MUT and CTNNB1-WT. Comparison of immune-related **(A)**, metabolic-related, and tumor-related signatures **(B)** between CTNNB1-MUT and CTNNB1-WT tumors in the TCGA-LIHC cohort. The results of GSEA in the TCGA-LIHC cohort relating to immune signaling **(C)** and immune-exhausted signaling **(D)**. The color of the curve corresponds to the font color of the pathway. The GSEA of hallmark gene sets was downloaded from the MSigDB and each run was performed with 1000 permutations. Enrichment results with significant differences between CTNNB1-MUT and CTNNB1-WT tumors are shown. **(E)** Comparison of IC50 value of drugs estimated by the pRRophetic algorithm between CTNNB1-MUT and CTNNB1-WT in the TCGA-LIHC cohort. *P < 0.05; **P < 0.01; ***P < 0.001; ****P < 0.0001.

Among the three immunophenotypes of solid tumors (immune inflammation, immune rejection, or immune depletion), tumors which display immune depletion have been shown in several studies to be the least sensitive to ICI treatment (34). To characterize the TIME of those with CTNNB1-MUT, we compared the immune cell fraction, expression of immune function-related molecules and immune-related signatures of the CTNNB1-MUT and CTNNB1-WT groups. CIBERSORT was used to evaluate the relative proportion of the 22 immune cell types, and we found a significant decrease in memory B cells, and a significant increase in M2-type macrophages in the CTNNB1-MUT group (P < 0.05; **Figure 5A**). IPS was used to evaluate the activity of the immune function, and we found the effector cells score (EC), a measure of activated CD8+/CD4+ T cells and TEM CD8+/CD4+ cell infiltration, to be significantly lower in the CTNNB1-MUT group than in the CTNNB1-WT group (P < 0.05; **Figure 5A**). Compared with CTNNB1-WT, CTNNB1-MUT had significantly more suppressor cells (SC) such as regulatory T cells (Tregs) and MDSCs (P < 0.05; **Figure 5A**). The ‘MCPcounter’ algorithm showed that the T cell score of patients in the CTNNB1-MUT group was significantly lower than of those in the CTNNB1-WT group (P < 0.05; **Figure 5A**). Similarly, the ‘quantiseq’ algorithm suggested that the proportion of B cells, M1-type macrophages, and dendritic cells (DCs) in the CTNNB1-MUT group was significantly lower than in the CTNNB1-WT group (P < 0.05; **Figure 5A**). The analysis of the immune-related molecule expression showed that immune checkpoint molecules (CD276, and HAVCR2) and immune stimulator molecules (CXCL12, CXCR4, IL6, TNF-related genes) were significantly lower in the CTNNB1-MUT group, compared to the CTNNB1-WT group (all P < 0.05; **Figure 5B**; **Table S3**). The activity of CTNNB1-MUT on immune-related pathways (cytokine receptors Li et al., TNF family members Li et al., type I IFN response Rooney et al., B cells Danaher et al. and cytotoxic cells Bindea et al.) was significantly lower than in the CTNNB1-WT group (all p < 0.05; **Figure 6A**; **Table S4**). In contrast to this, pathways which promote tumor growth and drug resistance (such as Wnt target, obesity acid elongation, drug metabolism by cytochrome P450 and fatty acid biosynthesis), showed significantly more activity in the CTNNB1-MUT group than in the CTNNB1-WT group (all P < 0.05; **Figure 6B**; **Table S4**). The association between CTNNB1 status and the pathways mentioned above were further verified using GSEA. This showed that the enrichment fraction of pathways which promote immune response in the CTNNB1-MUT group was significantly lower than that in the CTNNB1-WT group (ES < 0, P < 0.05; **Figure 6C**). At the same time, the enrichment fraction of pathways which promote immune depletion in the CTNNB1-MUT group was significantly higher than that in the CTNNB1-WT group (ES > 0, P< 0.05; **Figure 6D**).

### Guiding Role of CTNNB1 in Drug Sensitivity

To better guide the clinical treatment of patients with HCC, we constructed a ridge regression model to predict the IC50 values of 138 drugs for each patient in the TCGA-LIHC cohort. Additionally, we identified drugs which showed different sensitivities between the CTNNB1-MUT and CTNNB1-WT groups for subsequent analysis (P < 0.05). We found that the sensitivity of ERK MAPK signaling inhibitors (PLX-4720, PD0325901, Refametinib, Selumetinib), PI3K/mtor signaling inhibitors (PF-4708671 and AKT inhibitor VIII), RTK signaling inhibitor (Axitinib), Wnt signaling inhibitor (FH535), EGFR signaling inhibitor (Lapatinib), DNA replication inhibitor (Etoposide) was significantly higher in the CTNNB1-WT group than in the CTNNB1-MUT group (**Figure 6E**). This suggests that it is still necessary to explore whether a combination of these drugs and ICIs can further improve the prognosis of CTNNB-WT patients undergoing immunotherapy in the future.

## Discussion

In this study, we systematically screened HCC patients for all non-synonymous mutations and with mutation frequencies greater than 10% and found only CTNNB1-MUT to be a suitable independent prognostic factor for those receiving ICI treatment (based on a multivariate COX risk proportional regression model). We then added other variables (such as common clinical features) into our univariate and multivariate COX risk ratio regression models, which ruled out the potential bias of clinical features regarding the suitability of CTNNB1-MUT as an independent prognostic factor. To explore the mechanism behind CTNNB1-MUT and poor immunotherapy response in HCC patients we focused on immune cells, expression of immune-related molecules, and immune related signatures, as the TIME has been shown to play an important role. We found that the TIME of patients with CTNNB1-MUT is mainly one of immune depletion, which is reflected in the low proportion of activated immune cells, high proportion of depleted immune cells, low expression of immune stimulating molecules, low expression of immune checkpoint molecules, low activation of immunoactivation-related pathways, and high activation of tumor growth-promoting or drug resistance-related pathways. The above results suggest that CTTNB1-MUT can be used as a biomarker for patients with HCC undergoing immunotherapy, and can help clinicians to more accurately distinguish responders from non-responders.

An immunoinflammatory TIME is helpful in improving patients response to immunotherapy (11, 14, 35). In our study, T cells, B cells, M1-type macrophages and DCs were significantly enriched in the TIME of CTNNB1-WT HCC patients. On the contrary, M2-type macrophages with immunosuppression characteristics were significantly lower in patients with CTNNB1-WT than in those with CTTNB1-MUT. DCs, derived from myeloid cells, can cross-present tumor antigens to T lymphocytes in draining lymph nodes (36), and studies have shown that DC-AdCCL21 cells modified by the CCL21 gene have resulted in extensive monocyte infiltration and significant reduction in tumor load (37). Tumor vaccination with autologous DCs expressing CCL21 resulted in increased infiltration of CD8+T cells and increased expression of tumor PD-L1 (38). Tumor-associated macrophages (TAMs) are highly plastic and exhibit various phenotypes including M1 type (classical activation, proinflammatory response of anti-tumor activity) and M2 type (non-classical activation, angiogenesis promotion and immunosuppression of original tumor activity) (39). Tumor infiltrating T lymphocytes, especially CD4+ and CD8+T cells and their immunoregulatory cytokines play an adaptive immune role. CD8+T cells combine with T cell receptors to produce interferon-γ (IFN-γ), tumor necrosis factor (TNF) and granzyme B, which targets tumor cells and results in tumor cell clearance (40). However, tumors are able to inhibit the function of CD8+T cells in a number of ways. For example, Tregs have been shown to directly inhibit the anti-tumor effect of CD8+T cells (41). Additionally, Bruno et al. showed that tumor infiltrating B cells can present endogenous tumor antigen to CD4+ TILs, changing the CD4+ TILs phenotype *in vitro*, and that the activated tumor infiltrating B cells were related to the activated IFN-γ CD4+ T cell response (42). The above results suggest that a significant reduction in activated TILs and significant increase in depleted immune cells may be one of the reasons for a less favorable prognosis in patients with CTNNB1-MUT who are receiving ICIs.

In addition to a TIME which displays immune exhaustion, metabolic reprogramming in the TIME also has some influence on anti-tumor activity (43, 44). Two-way regulation takes place between tumor cells and immune cells in the TIME in the following ways: 1) tumor cells recruit and regulate the behavior of immune cells by secreting growth factors and cytokines; 2) the interaction between tumor cells and immune cells can break through the internal balance of the body, mobilizing the internal and external resources of cells, creating a suitable TIME for their own growth, and affecting the response of tumor cells to immunotherapy (43). Activated neutrophils and M1 macrophages rely mainly on the glycolytic pathway for energy supply, while Tregs and M2 macrophages mainly utilize oxidative phosphorylation of fatty acid β (44–46). As the high rate of cholesterol esterification which occurs in tumors can damage the reaction of T cells, inhibiting this esterification reaction by increasing the concentration of cholesterol in CD8+T plasma membranes may help to promote the proliferation and improve the effector function of CD8+T cells (47). In addition to lipid metabolism pathways, the WNT signaling pathway can also inhibit the activation of tumor infiltrating immune cells, promote the apoptosis of T cells, inhibit antigen treatment and degree, and finally affect the response of tumor cells to immunotherapy (48). In this study we found, using PCA, z-score and GSEA, that the activity of immune activation and response pathways was significantly down-regulated in patients with CTTNB1-MUT. However, some pathways, such as those related to immune depletion or the promotion of tumor growth, were significantly up-regulated in patients with CTNNB1-MUT, perhaps explaining why HCC patients with CTNNB1-MUT have a significantly worse immunotherapy prognosis.

It is important to note that this research still has some limitations. First, the HCC cohort with mutation and immunotherapy prognosis data is very small, coming from only one study (Harding et al.). To solve this problem, we have prospectively recruited HCC patients receiving ICIs to further verify the relationship between CTNNB1-MUT and immunotherapy response in the future. Second, the lack of a functional test is another major weakness of this study. In the future, we hope to further explore the potential mechanism between CTNNB1-MUT and the prognosis of immunotherapy through both cell and animal experiments. Third, the mutation data in the Harding-HCC cohort is from targeted sequencing, which unlike WES, includes only the genes most common in clinical practice.

## Conclusions

In this study, we found that CTNNB1-MUT may be a suitable biomarker for patients with HCC undergoing immunotherapy, and can be used to more accurately distinguish patients less likely to benefit from ICIs. The TIME of CTNNB1-MUT HCC shows immune depletion which is manifested by significantly reduced activated TILs, significantly increased immunosuppressive immune cells, and significantly reduced expression of immunostimulating molecules. Also evident is low activity in immune activation pathways and high activity in immune depletion pathways.

## Data Availability Statement

The original contributions presented in the study are included in the article/**Supplementary Material**. Further inquiries can be directed to the corresponding author.

## Ethics Statement

Ethical review and approval were not required for the study on human participants in accordance with the local legislation and institutional requirements. Written informed consent for participation was not required for this study in accordance with the national legislation and the institutional requirements.

## Author Contributions

Conceptualization, WZ. Formal analysis, LC. Visualization, LC. Writing–original draft, QZ and JL. Writing–review & editing, LC, QZ, and JL. All authors contributed to the article and approved the submitted version.

## Funding

The article was supported by the Project of the Natural Science Foundation of China, No. 81860117.

## Conflict of Interest

The authors declare that the research was conducted in the absence of any commercial or financial relationships that could be construed as a potential conflict of interest.

## Publisher’s Note

All claims expressed in this article are solely those of the authors and do not necessarily represent those of their affiliated organizations, or those of the publisher, the editors and the reviewers. Any product that may be evaluated in this article, or claim that may be made by its manufacturer, is not guaranteed or endorsed by the publisher.

## Supplementary Material

Supplementary Table 1 The clinical characteristics of the HCC patients in the Harding-HCC.

Supplementary Table 2 The TCGA-LIHC cohorts.

Supplementary Table 3 The result of the **Figure 5B**.

Supplementary Table 4 The result of the **Figures 6A, B**.

Supplementary Figure 1 The Top 20 mutated genes in the Harding-HCC **(A)** and the TCGA-LIHC **(B)** cohorts. (*P < 0.05; **P < 0.01; ***P < 0.001; ****P < 0.0001; Fisher’s exact test).
