# Supplementary material for: CTNNB1 Alternation Is a Potential Biomarker for Immunotherapy Prognosis in Patients With Hepatocellular Carcinoma
Source: Front Immunol. 2021 Oct 28;12:759565. doi: 10.3389/fimmu.2021.759565 (PMC8581472; doi:10.3389/fimmu.2021.759565)
Supplement: Supplementary Table 1 — The clinical characteristics of the HCC patients in the Harding-HCC. [file DataSheet_1.pdf]

	CTNNB1-MUT (N=7)	CTNNB1-WT (N=20)	Overall (N=27)
**Gender**			
Female	3 (42.9%)	8 (40.0%)	11 (40.7%)
Male	4 (57.1%)	12 (60.0%)	16 (59.3%)
**Race**			
Asian	2 (28.6%)	5 (25.0%)	7 (25.9%)
Black	1 (14.3%)	0 (0%)	1 (3.7%)
Unknown	0 (0%)	1 (5.0%)	1 (3.7%)
White	4 (57.1%)	14 (70.0%)	18 (66.7%)
**HCV**			
No	5 (71.4%)	17 (85.0%)	22 (81.5%)
Yes	2 (28.6%)	3 (15.0%)	5 (18.5%)
**HBV**			
No	6 (85.7%)	14 (70.0%)	20 (74.1%)
Yes	1 (14.3%)	6 (30.0%)	7 (25.9%)
**Response**			
CR	0 (0%)	1 (5.0%)	1 (3.7%)
PD	7 (100%)	8 (40.0%)	15 (55.6%)
PR	0 (0%)	2 (10.0%)	2 (7.4%)
SD	0 (0%)	9 (45.0%)	9 (33.3%)
