# Supplementary material for: CTNNB1 Alternation Is a Potential Biomarker for Immunotherapy Prognosis in Patients With Hepatocellular Carcinoma
Source: Front Immunol. 2021 Oct 28;12:759565. doi: 10.3389/fimmu.2021.759565 (PMC8581472; doi:10.3389/fimmu.2021.759565)
Supplement: Supplementary Table 2 — The TCGA-LIHC cohorts. [file DataSheet_2.pdf]

	CTNNB1-MUT (N=87)	CTNNB1-WT (N=274)	Overall (N=361)
**Gender**			
Female	13 (14.9%)	106 (38.7%)	119 (33.0%)
Male	74 (85.1%)	168 (61.3%)	242 (67.0%)
**TNM Stage**			
I	38 (43.7%)	131 (47.8%)	169 (46.8%)
II	22 (25.3%)	60 (21.9%)	82 (22.7%)
III	24 (27.6%)	60 (21.9%)	84 (23.3%)
IV	0 (0%)	5 (1.8%)	5 (1.4%)
Unknown	3 (3.4%)	18 (6.6%)	21 (5.8%)
**Ethnicity**			
hispanic or latino	4 (4.6%)	14 (5.1%)	18 (5.0%)
not hispanic or latino	82 (94.3%)	244 (89.1%)	326 (90.3%)
Unknown	1 (1.1%)	16 (5.8%)	17 (4.7%)
**Age**			
Mean (SD)	62.1 (11.1)	58.2 (14.1)	59.1 (13.5)
Median [Min, Max]	65.0 [23.0, 83.0]	60.0 [16.0, 90.0]	61.0 [16.0, 90.0]
