# Supplementary material for: CTNNB1 Alternation Is a Potential Biomarker for Immunotherapy Prognosis in Patients With Hepatocellular Carcinoma
Source: Front Immunol. 2021 Oct 28;12:759565. doi: 10.3389/fimmu.2021.759565 (PMC8581472; doi:10.3389/fimmu.2021.759565)
Supplement: Supplementary Table 3 — The result of the Figure 5B . [file DataSheet_3.pdf]

Gene	logFC	AveExpr	t	P.Value	adj.P.Val	B	Label	Reference
CD276	-3.605421	10.931	-4.2	3E-05	0.00141	2.05939153	Immune Checkpoint	doi: 10.1016/j.immuni.2018.03.023. doi: 10.1016/j.cell.2014.12.033.
HAVCR2	-1.135174	1.9796	-2.2	0.0263	0.09298	-4.030915033	Immune Checkpoint	doi: 10.1016/j.immuni.2018.03.023. doi: 10.1016/j.cell.2014.12.033.
VTCN1	-3.306806	2.6909	-1.5	0.1221	0.28078	-5.278253497	Immune Checkpoint	doi: 10.1016/j.immuni.2018.03.023. doi: 10.1016/j.cell.2014.12.033.
CD274	-0.157124	0.5242	-1.3	0.1839	0.36787	-5.584292135	Immune Checkpoint	doi: 10.1016/j.immuni.2018.03.023. doi: 10.1016/j.cell.2014.12.033.
CTLA4	-0.125768	0.582	-1.1	0.2625	0.44446	-5.833973691	Immune Checkpoint	doi: 10.1016/j.immuni.2018.03.023. doi: 10.1016/j.cell.2014.12.033.
LAG3	-0.453774	1.6904	-1.1	0.2646	0.44446	-5.83951611	Immune Checkpoint	doi: 10.1016/j.immuni.2018.03.023. doi: 10.1016/j.cell.2014.12.033.
PDCD1L(	-0.293741	0.6267	-1.1	0.2724	0.44446	-5.859089425	Immune Checkpoint	doi: 10.1016/j.immuni.2018.03.023. doi: 10.1016/j.cell.2014.12.033.
PDCD1	-0.396354	1.3206	-0.9	0.3664	0.51301	-6.049455927	Immune Checkpoint	doi: 10.1016/j.immuni.2018.03.023. doi: 10.1016/j.cell.2014.12.033.
TIGIT	-0.079653	0.4836	-0.7	0.509	0.6532	-6.235392207	Immune Checkpoint	doi: 10.1016/j.immuni.2018.03.023. doi: 10.1016/j.cell.2014.12.033.
IDO1	-0.061632	1.2239	-0.2	0.8721	0.90357	-6.436283217	Immune Checkpoint	doi: 10.1016/j.immuni.2018.03.023. doi: 10.1016/j.cell.2014.12.033.

CD96	-0.221907	0.6137	-1.9	0.0586	0.16071	-4.696907636	Immunoinhibitor	doi: 10.1016/j.immuni.2018.03.023. doi: 10.1016/j.cell.2014.12.033.
CSF1R	-1.693757	5.4857	-1.8	0.0747	0.19644	-4.893632787	Immunoinhibitor	doi: 10.1016/j.immuni.2018.03.023. doi: 10.1016/j.cell.2014.12.033.
CD160	-0.040236	0.1326	-1.7	0.0998	0.24816	-5.122406676	Immunoinhibitor	doi: 10.1016/j.immuni.2018.03.023. doi: 10.1016/j.cell.2014.12.033.
IL10	-0.065681	0.1387	-1.4	0.1657	0.36298	-5.507985401	Immunoinhibitor	doi: 10.1016/j.immuni.2018.03.023. doi: 10.1016/j.cell.2014.12.033.
KIR2DL3	-0.008355	0.0214	-1.3	0.1928	0.37745	-5.61839366	Immunoinhibitor	doi: 10.1016/j.immuni.2018.03.023. doi: 10.1016/j.cell.2014.12.033.
ADORA2/	0.028643	0.4714	0.65	0.5161	0.6532	-6.242400861	Immunoinhibitor	doi: 10.1016/j.immuni.2018.03.023. doi: 10.1016/j.cell.2014.12.033.
KIR2DL1	-0.002009	0.023	-0.3	0.7969	0.87276	-6.416529546	Immunoinhibitor	doi: 10.1016/j.immuni.2018.03.023. doi: 10.1016/j.cell.2014.12.033.
CD244	0.0133759	0.3527	0.2	0.8431	0.89153	-6.429788296	Immunoinhibitor	doi: 10.1016/j.immuni.2018.03.023. doi: 10.1016/j.cell.2014.12.033.
BTLA	-0.007226	0.1151	-0.2	0.8741	0.90357	-6.436686765	Immunoinhibitor	doi: 10.1016/j.immuni.2018.03.023. doi: 10.1016/j.cell.2014.12.033.
KDR	-0.05791	4.2236	-0.1	0.8891	0.90887	-6.439460682	Immunoinhibitor	doi: 10.1016/j.immuni.2018.03.023. doi: 10.1016/j.cell.2014.12.033.

ENTPD1	-0.461203	1.4707	-4.3	2E-05	0.00141	2.476013644	Immunostimulator	doi: 10.1016/j.immuni.2018.03.023. doi: 10.1016/j.cell.2014.12.033.
TNFSF15	-0.302242	0.3448	-3.8	0.0002	0.0051	0.492323788	Immunostimulator	doi: 10.1016/j.immuni.2018.03.023. doi: 10.1016/j.cell.2014.12.033.
TNFRSF1	-2.394059	9.3452	-3.6	0.0003	0.00791	-0.174877833	Immunostimulator	doi: 10.1016/j.immuni.2018.03.023. doi: 10.1016/j.cell.2014.12.033.
CXCL12	-3.682184	7.2507	-3.3	0.0011	0.01726	-1.254875949	Immunostimulator	doi: 10.1016/j.immuni.2018.03.023. doi: 10.1016/j.cell.2014.12.033.
CXCR4	-4.977087	12.268	-3.1	0.0019	0.02132	-1.705215947	Immunostimulator	doi: 10.1016/j.immuni.2018.03.023. doi: 10.1016/j.cell.2014.12.033.
TMEM17[illegible]	-1.823264	3.7882	-2.9	0.0044	0.03535	-2.480088982	Immunostimulator	doi: 10.1016/j.immuni.2018.03.023. doi: 10.1016/j.cell.2014.12.033.
TNFSF13	-0.449697	1.2949	-2.8	0.005	0.03535	-2.589260587	Immunostimulator	doi: 10.1016/j.immuni.2018.03.023. doi: 10.1016/j.cell.2014.12.033.
TNFRSF2	-0.800989	1.7501	-2.8	0.0054	0.03535	-2.655387797	Immunostimulator	doi: 10.1016/j.immuni.2018.03.023. doi: 10.1016/j.cell.2014.12.033.
ULBP1	-0.097112	0.1239	-2.7	0.008	0.04852	-3.010036103	Immunostimulator	doi: 10.1016/j.immuni.2018.03.023. doi: 10.1016/j.cell.2014.12.033.
PVR	-2.78003	18.511	-2.6	0.0088	0.04852	-3.085208245	Immunostimulator	doi: 10.1016/j.immuni.2018.03.023. doi: 10.1016/j.cell.2014.12.033.

TMIGD2	-0.102113	0.2298	-2.6	0.009	0.04852	-3.104730941	Immunostimulator	doi: 10.1016/j.immuni.2018.03.023. doi: 10.1016/j.cell.2014.12.033.
TNFSF13	-0.695419	1.3793	-2.5	0.0123	0.05456	-3.377389028	Immunostimulator	doi: 10.1016/j.immuni.2018.03.023. doi: 10.1016/j.cell.2014.12.033.
IL6	-0.245011	0.2854	-2.5	0.0125	0.05456	-3.390859784	Immunostimulator	doi: 10.1016/j.immuni.2018.03.023. doi: 10.1016/j.cell.2014.12.033.
TNFRSF8	-0.056113	0.12	-2.5	0.0136	0.0569	-3.467498331	Immunostimulator	doi: 10.1016/j.immuni.2018.03.023. doi: 10.1016/j.cell.2014.12.033.
TNFRSF1	-0.547105	1.2615	-2.3	0.0232	0.08909	-3.926769111	Immunostimulator	doi: 10.1016/j.immuni.2018.03.023. doi: 10.1016/j.cell.2014.12.033.
CD86	-0.483571	1.4448	-2.2	0.0262	0.09298	-4.02724683	Immunostimulator	doi: 10.1016/j.immuni.2018.03.023. doi: 10.1016/j.cell.2014.12.033.
TNFSF4	-0.473939	1.0387	-2.1	0.0348	0.1103	-4.266516023	Immunostimulator	doi: 10.1016/j.immuni.2018.03.023. doi: 10.1016/j.cell.2014.12.033.
MICB	-0.429502	1.7603	-1.9	0.0521	0.15515	-4.601602897	Immunostimulator	doi: 10.1016/j.immuni.2018.03.023. doi: 10.1016/j.cell.2014.12.033.
IL2RA	-0.20218	0.4364	-1.9	0.0523	0.15515	-4.604250588	Immunostimulator	doi: 10.1016/j.immuni.2018.03.023. doi: 10.1016/j.cell.2014.12.033.
TNFSF14	-1.591447	4.4478	-1.9	0.0542	0.15578	-4.633568622	Immunostimulator	doi: 10.1016/j.immuni.2018.03.023. doi: 10.1016/j.cell.2014.12.033.

TNFSF18	-0.040784	0.1439	-1.7	0.0965	0.24658	-5.095971891	Immunostimulator	doi: 10.1016/j.immuni.2018.03.023. doi: 10.1016/j.cell.2014.12.033.
CD80	-0.055249	0.1393	-1.6	0.1167	0.2754	-5.243964247	Immunostimulator	doi: 10.1016/j.immuni.2018.03.023. doi: 10.1016/j.cell.2014.12.033.
CD40LG	-0.131048	0.4549	-1.3	0.1815	0.36787	-5.574660721	Immunostimulator	doi: 10.1016/j.immuni.2018.03.023. doi: 10.1016/j.cell.2014.12.033.
CD48	-0.660628	2.1996	-1.3	0.2029	0.38889	-5.654890627	Immunostimulator	doi: 10.1016/j.immuni.2018.03.023. doi: 10.1016/j.cell.2014.12.033.
TNFSF9	-0.160078	0.5503	-1.2	0.2352	0.42699	-5.758875066	Immunostimulator	doi: 10.1016/j.immuni.2018.03.023. doi: 10.1016/j.cell.2014.12.033.
TNFRSF4	-0.265847	2.1707	-1.2	0.2367	0.42699	-5.763234321	Immunostimulator	doi: 10.1016/j.immuni.2018.03.023. doi: 10.1016/j.cell.2014.12.033.
LTA	-0.070245	0.2634	-1.1	0.2663	0.44446	-5.84380352	Immunostimulator	doi: 10.1016/j.immuni.2018.03.023. doi: 10.1016/j.cell.2014.12.033.
ICOS	-0.069263	0.2777	-1.1	0.2754	0.44446	-5.866250131	Immunostimulator	doi: 10.1016/j.immuni.2018.03.023. doi: 10.1016/j.cell.2014.12.033.
CD70	-0.243254	0.3281	-1.1	0.2878	0.45644	-5.89549161	Immunostimulator	doi: 10.1016/j.immuni.2018.03.023. doi: 10.1016/j.cell.2014.12.033.
RAET1E	-0.010296	0.0501	-1	0.3245	0.49752	-5.973482825	Immunostimulator	doi: 10.1016/j.immuni.2018.03.023. doi: 10.1016/j.cell.2014.12.033.

CD28	-0.065273	0.4294	-0.9	0.3493	0.51301	-6.020002084	Immunostimulator	doi: 10.1016/j.immuni.2018.03.023. doi: 10.1016/j.cell.2014.12.033.
HHLA2	-0.057782	0.107	-0.9	0.3499	0.51301	-6.020995746	Immunostimulator	doi: 10.1016/j.immuni.2018.03.023. doi: 10.1016/j.cell.2014.12.033.
CXCR3	-0.204578	1.0782	-0.9	0.3572	0.51301	-6.033731249	Immunostimulator	doi: 10.1016/j.immuni.2018.03.023. doi: 10.1016/j.cell.2014.12.033.
TNFRSF1	-0.019374	0.0655	-0.7	0.4659	0.62847	-6.188544028	Immunostimulator	doi: 10.1016/j.immuni.2018.03.023. doi: 10.1016/j.cell.2014.12.033.
CD27	-0.330669	1.7362	-0.7	0.4748	0.62847	-6.198827576	Immunostimulator	doi: 10.1016/j.immuni.2018.03.023. doi: 10.1016/j.cell.2014.12.033.
TNFRSF1	-0.060922	0.2146	-0.6	0.5509	0.68493	-6.274777742	Immunostimulator	doi: 10.1016/j.immuni.2018.03.023. doi: 10.1016/j.cell.2014.12.033.
KLRK1	-0.00767	0.0623	-0.5	0.5862	0.71274	-6.303849749	Immunostimulator	doi: 10.1016/j.immuni.2018.03.023. doi: 10.1016/j.cell.2014.12.033.
MICA	-0.261973	6.1124	-0.5	0.5965	0.71274	-6.311716579	Immunostimulator	doi: 10.1016/j.immuni.2018.03.023. doi: 10.1016/j.cell.2014.12.033.
BTNL2	-0.001559	0.0047	-0.4	0.6679	0.78241	-6.358836022	Immunostimulator	doi: 10.1016/j.immuni.2018.03.023. doi: 10.1016/j.cell.2014.12.033.
CD40	-0.334554	7.4951	-0.4	0.6719	0.78241	-6.361093673	Immunostimulator	doi: 10.1016/j.immuni.2018.03.023. doi: 10.1016/j.cell.2014.12.033.

NT5E	0.3907085	10.787	0.32	0.7475	0.83862	-6.398203272	Immunostimulator	doi: 10.1016/j.immuni.2018.03.023. doi: 10.1016/j.cell.2014.12.033.
CXCL10	2.5114585	29.357	0.28	0.7791	0.86359	-6.410450098	Immunostimulator	doi: 10.1016/j.immuni.2018.03.023. doi: 10.1016/j.cell.2014.12.033.
CXCL9	0.8489056	13.627	0.21	0.8319	0.89153	-6.426919373	Immunostimulator	doi: 10.1016/j.immuni.2018.03.023. doi: 10.1016/j.cell.2014.12.033.
TNFRSF1	0.0152555	0.3334	0.1	0.9185	0.92862	-6.443857377	Immunostimulator	doi: 10.1016/j.immuni.2018.03.023. doi: 10.1016/j.cell.2014.12.033.
ICOSLG	2.801E-05	0.1408	0	0.9989	0.99886	-6.448981335	Immunostimulator	doi: 10.1016/j.immuni.2018.03.023. doi: 10.1016/j.cell.2014.12.033.
HLA-A	59.469818	453.1	1.41	0.1606	0.36042	-5.484952166	MHC-classical_class-I	doi: 10.1016/j.immuni.2018.03.023. doi: 10.1016/j.cell.2014.12.033.
HLA-B	83.172867	524.72	1.38	0.1697	0.36304	-5.525411638	MHC-classical_class-I	doi: 10.1016/j.immuni.2018.03.023. doi: 10.1016/j.cell.2014.12.033.
TAP2	0.6007051	4.4811	1.35	0.1777	0.36787	-5.559215109	MHC-classical_class-I	doi: 10.1016/j.immuni.2018.03.023. doi: 10.1016/j.cell.2014.12.033.
TAPBP	-3.439393	62.588	-0.9	0.368	0.51301	-6.052110435	MHC-classical_class-I	doi: 10.1016/j.immuni.2018.03.023. doi: 10.1016/j.cell.2014.12.033.
TAP1	-0.927373	17.021	-0.3	0.7399	0.83862	-6.395008741	MHC-classical_class-I	doi: 10.1016/j.immuni.2018.03.023. doi: 10.1016/j.cell.2014.12.033.

HLA-DQA	-2.530961	7.1165	-1.9	0.0594	0.16071	-4.708335895	MHC-classical_class-II	doi: 10.1016/j.immuni.2018.03.023. doi: 10.1016/j.cell.2014.12.033.
HLA-DQA	-2.894999	7.8594	-1.6	0.1038	0.25136	-5.153183828	MHC-classical_class-II	doi: 10.1016/j.immuni.2018.03.023. doi: 10.1016/j.cell.2014.12.033.
HLA-DQB	2.4039533	13.385	1.01	0.3122	0.48684	-5.948776209	MHC-classical_class-II	doi: 10.1016/j.immuni.2018.03.023. doi: 10.1016/j.cell.2014.12.033.
HLA-DPB	-6.94972	47.654	-0.9	0.3536	0.51301	-6.027466273	MHC-classical_class-II	doi: 10.1016/j.immuni.2018.03.023. doi: 10.1016/j.cell.2014.12.033.
HLA-DRB	24.329811	176.79	0.82	0.4129	0.56695	-6.120592525	MHC-classical_class-II	doi: 10.1016/j.immuni.2018.03.023. doi: 10.1016/j.cell.2014.12.033.
HLA-DPA	-1.075198	20.153	-0.3	0.7454	0.83862	-6.397324405	MHC-classical_class-II	doi: 10.1016/j.immuni.2018.03.023. doi: 10.1016/j.cell.2014.12.033.
HLA-DRA	-9.012801	256.08	-0.2	0.8428	0.89153	-6.429729059	MHC-classical_class-II	doi: 10.1016/j.immuni.2018.03.023. doi: 10.1016/j.cell.2014.12.033.
HLA-G	0.4945124	2.8354	1.19	0.2336	0.42699	-5.754115526	MHC-non-class_class-I	doi: 10.1016/j.immuni.2018.03.023. doi: 10.1016/j.cell.2014.12.033.
HLA-F	3.5937516	23.989	1.17	0.244	0.43166	-5.784147468	MHC-non-class_class-I	doi: 10.1016/j.immuni.2018.03.023. doi: 10.1016/j.cell.2014.12.033.
HLA-DOB	-0.284163	1.3351	-0.7	0.4782	0.62847	-6.202621496	MHC-non-class_class-II	doi: 10.1016/j.immuni.2018.03.023. doi: 10.1016/j.cell.2014.12.033.

HLA-DOA	-0.414235	2.9434	-0.6	0.5183	0.6532	-6.244607133	MHC-non-class_class-II	doi: 10.1016/j.immuni.2018.03.023. doi: 10.1016/j.cell.2014.12.033.
HLA-DME	-0.410406	4.6004	-0.5	0.5958	0.71274	-6.311192624	MHC-non-class_class-II	doi: 10.1016/j.immuni.2018.03.023. doi: 10.1016/j.cell.2014.12.033.
