# Supplementary material for: CTNNB1 Alternation Is a Potential Biomarker for Immunotherapy Prognosis in Patients With Hepatocellular Carcinoma
Source: Front Immunol. 2021 Oct 28;12:759565. doi: 10.3389/fimmu.2021.759565 (PMC8581472; doi:10.3389/fimmu.2021.759565)
Supplement: Supplementary Table 4 — The result of the Figures 6A, B . [file DataSheet_4.pdf]

Pathways	Algorithm	Pvalue	MUT-WT	Function	Reference
WNT_target_PCA	PCA	1.49E-05	MUT-UP****	Promote tumor growth and drug resistance	doi: 10.3389/fimmu.2021.687975
Fatty_Acid_Elongation_PCA	PCA	9.35E-05	MUT-UP****	Promote tumor growth and drug resistance	doi: 10.3389/fimmu.2021.687975
Biosynthesis_of_Unsaturated_Fatty_Acids_PCA	PCA	6.02E-05	MUT-UP****	Promote tumor growth and drug resistance	doi: 10.3389/fimmu.2021.687975
Drug_Metabolism_by_Cytochrome_P450_PCA	PCA	1.33E-14	MUT-UP****	Promote tumor growth and drug resistance	doi: 10.3389/fimmu.2021.687975
Drug_Metabolism_by_other_enzymes_PCA	PCA	6.80E-14	MUT-UP****	Promote tumor growth and drug resistance	doi: 10.3389/fimmu.2021.687975
Immune_Checkpoint_PCA	PCA	0.005025077	MUT-Down**	Promote tumor growth and drug resistance	doi: 10.3389/fimmu.2021.687975
Fatty_Acid_Biosynthesis_zscore	zscore	0.002388137	MUT-UP**	Promote tumor growth and drug resistance	doi: 10.3389/fimmu.2021.687975
DDR_PCA	PCA	0.032062055	MUT-Down*	Immune-related	doi: 10.3389/fimmu.2021.687975

Cytokine_Receptors_Li_et_al_PCA	PCA	3.03E-07	MUT-Down****	Immune-related	doi: 10.3389/fimmu.2021.687975
TNF_Family_Members_Li_et_al_PCA	PCA	4.66E-06	MUT-Down****	Immune-related	doi: 10.3389/fimmu.2021.687975
Type_I_IFN_Reponse_Rooney_et_al_PCA	PCA	0.000198865	MUT-Down***	Immune-related	doi: 10.3389/fimmu.2021.687975
B_cells_Danaher_et_al_PCA	PCA	0.000724846	MUT-Down***	Immune-related	doi: 10.3389/fimmu.2021.687975
pDCs_Rooney_et_al_PCA	PCA	0.007961156	MUT-Down**	Immune-related	doi: 10.3389/fimmu.2021.687975
Co_stimulation_T_cell_Rooney_et_al_PCA	PCA	0.001004661	MUT-Down**	Immune-related	doi: 10.3389/fimmu.2021.687975
NK_cells_Danaher_et_al_PCA	PCA	0.001488431	MUT-Down**	Immune-related	doi: 10.3389/fimmu.2021.687975
T_cells_Danaher_et_al_PCA	PCA	0.003240098	MUT-Down**	Immune-related	doi: 10.3389/fimmu.2021.687975
CD8_Rooney_et_al_PCA	PCA	0.025298649	MUT-Down*	Immune-related	doi: 10.3389/fimmu.2021.687975
NK_CD56dim_cells_Danaher_et_al_PCA	PCA	0.039282863	MUT-Down*	Immune-related	doi: 10.3389/fimmu.2021.687975
Cytotoxic_cells_Bindea_et_al_PCA	PCA	0.049477667	MUT-Down*	Immune-related	doi: 10.3389/fimmu.2021.687975
