# Supplementary figures and images for: CTNNB1 Alternation Is a Potential Biomarker for Immunotherapy Prognosis in Patients With Hepatocellular Carcinoma
Source: Front Immunol. 2021 Oct 28;12:759565. doi: 10.3389/fimmu.2021.759565 (PMC8581472; doi:10.3389/fimmu.2021.759565)

A

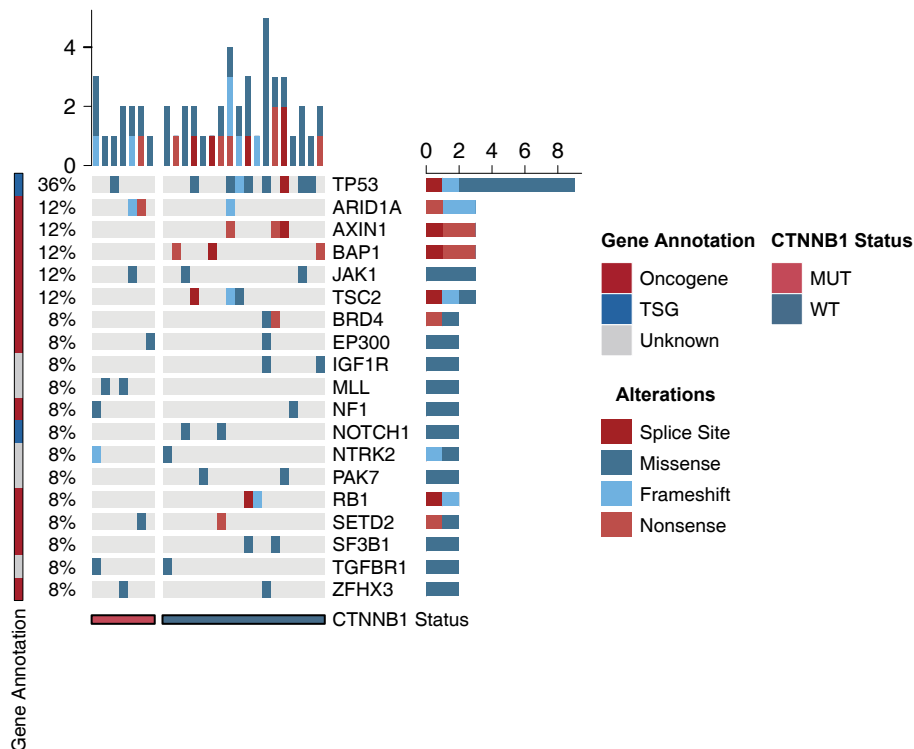

B

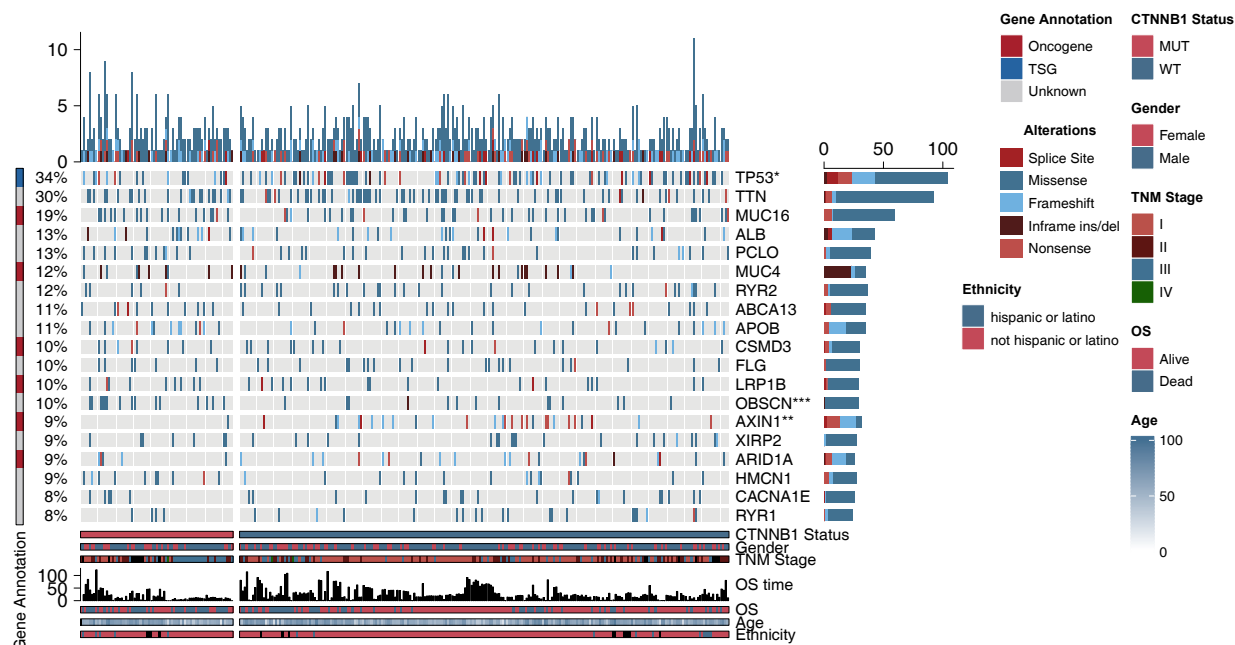

Supplement: Supplementary Figure 1 — The Top 20 mutated genes in the Harding-HCC (A) and the TCGA-LIHC (B) cohorts. (*P < 0.05; **P < 0.01; ***P < 0.001; ****P < 0.0001; Fisher’s exact test). [file Image_1.pdf]
